# Supplementary material for: Nanosecond pulsed electric fields increase antibiotic susceptibility in methicillin-resistant Staphylococcus aureus
Source: Microbiol Spectr. 2023 Dec 4;12(1):e02992-23. doi: 10.1128/spectrum.02992-23 (PMC10783032; doi:10.1128/spectrum.02992-23)
Supplement: Supplemental figures — Fig. S1 to S3. [file spectrum.02992-23-s0001.pdf]

**Supplementary Material**

**Figure S1**

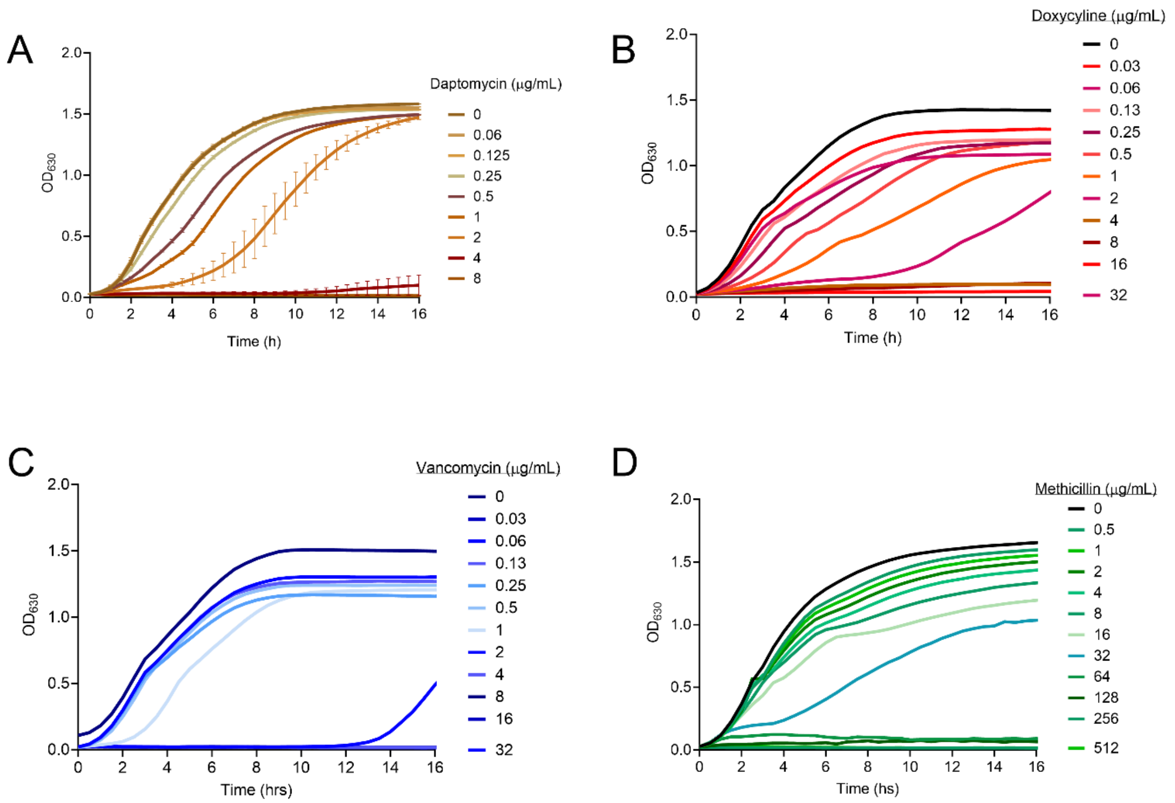


**Figure S1. Growth inhibition of MRSA cultures by antibiotics.** MRSA cultures were diluted in sterile 96-well plates using a 1:20 ratio in LB broth containing a range of indicated concentrations of (A) daptomycin, (B) doxycycline, (C) vancomycin, or (D) methicillin. Plates were incubated at 37.0 ºC for 24 h in a microplate reader, which was set to constantly shake at medium intensity and the optical density was recorded at 630 nm every 30 min.

Figure S2

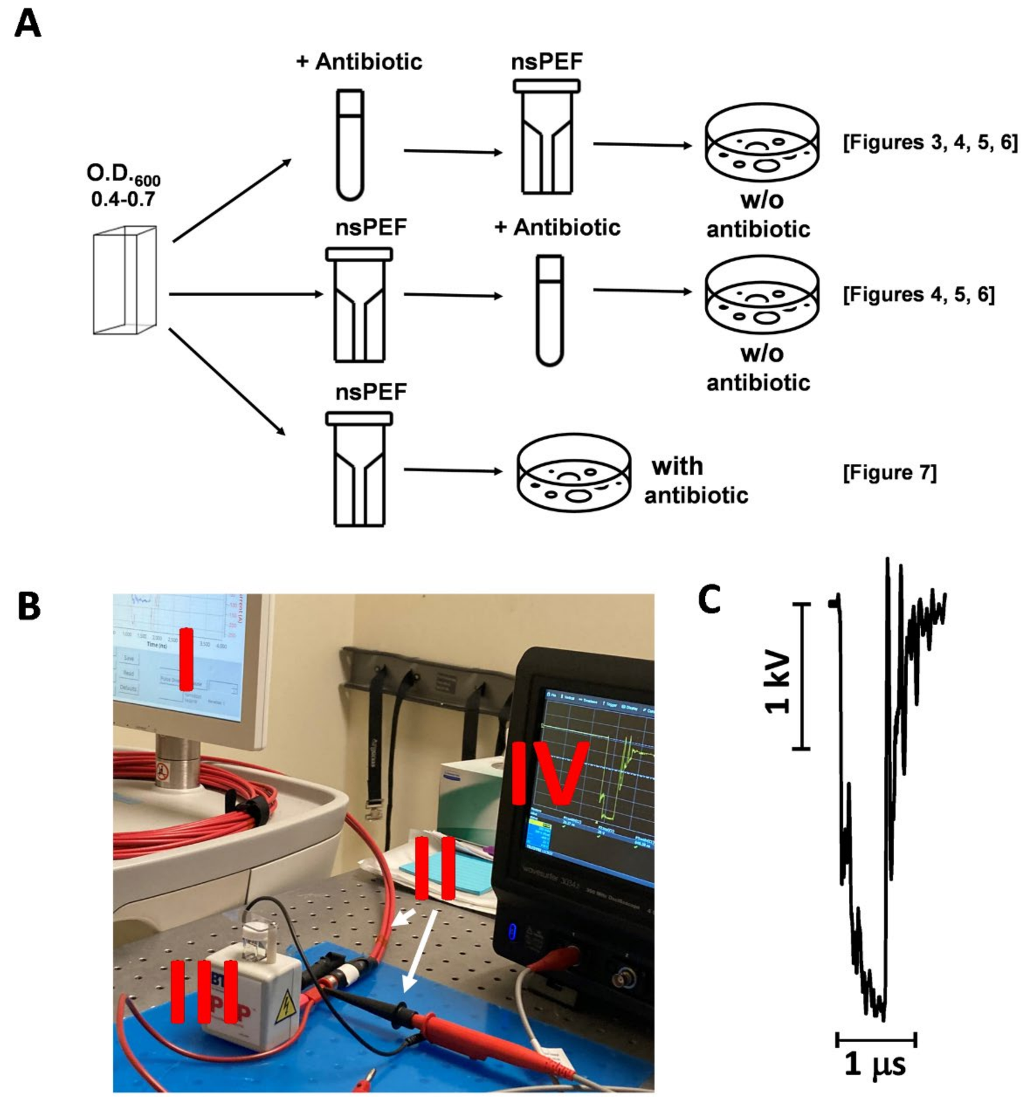


**Figure S2. Diagram of the experimental workflow (A), nsPEF exposure system (B), and waveform (C)**. The diagram in (A) shows that MRSA cultures in exponential growth phase ($OD_{600}$ = 0.4–0.7) were either transiently incubated with antibiotics for 90 minutes pre- or post-nsPEF treatment and plated on plates without antibiotics or, for prolonged antibiotic exposure, treated with nsPEF and then plated on plates with antibiotics. The picture in (B) shows the generator (I), the cable with the oscilloscope probe (II, white arrows) connected before the

cuvette holder (III) and the oscilloscope in the back (IV). (C) is a representative 600 ns waveform.

**Figure S3**

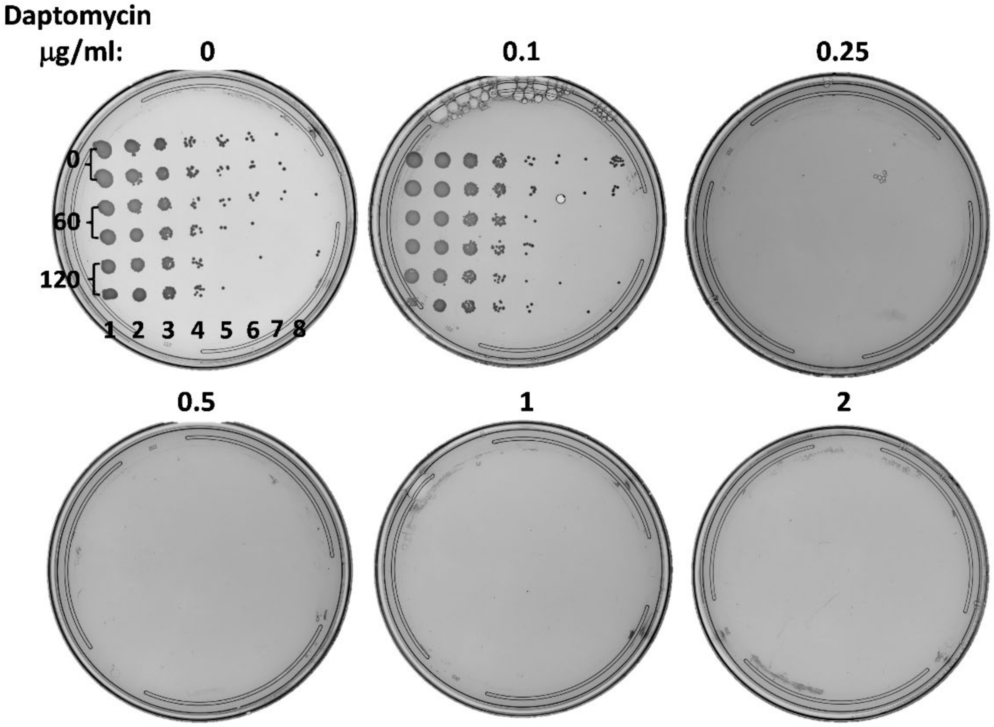


**Figure S3. Effect of combining nsPEF with prolonged exposure to daptomycin**. Exponential phase MRSA cultures were treated with 0, 60, or 120 pulses (600 ns, 28 kV/cm, 1 Hz). The treated samples underwent a serial dilution using LB broth in a sterile 96-well plate. A sterile replica plater was used to plate the samples on LB agar plates containing the indicated concentrations of daptomycin. The plates were incubated at 37.0 ºC for 24 h before they were scanned using a ChemiDoc MP Imaging System. Shown are the representative images of 8 serial 10-fold dilutions.
